# Strong correlation between optical properties and mechanism in deficiency of normalized self-assembly ZnO nanorods

**DOI:** 10.1038/s41598-018-37601-8

**Published:** 2019-01-29

**Authors:** Feng-Ming Chang, Sanjaya Brahma, Jing-Heng Huang, Zong-Zhe Wu, Kuang-Yao Lo

**Affiliations:** Department of Physics, National Cheng Kung University, Tainan, 701 Taiwan

## Abstract

Although, post annealing is an efficient way to annihilate/restructure deficiencies in self-assembly (SA) ZnO nanorods (ZNRs), the detailed investigation about the surface properties of annealed SA-ZNRs is a long standing issue and the major discrepancy is mainly due to single step annealing. We demonstrate the strategic two step annealing process to create reliable structural configuration in SA-ZNRs during the first round of annealing at 800 °C in vacuum (VA process), and create intrinsic defects in the second step of annealing in oxygen rich atmosphere (OA process) to correlate the formation of the defects related to green/orange-red emission. SA-ZNRs annealed in VA-OA processes reveal positive correlations between the oxygen flow rate and formation of oxygen interstitials (O_i_) and zinc vacancies (V_Zn_). The OA-VA processes exhibit the relation of residual O_i_ and additional V_o_. According to VA-OA and OA-VA processes, we propose that the green emission in ZnO annealed in oxygen poor/rich condition is mainly due to the formation of V_o_/V_Zn_ and annealing at oxygen rich condition creates O_i_ that lead to strong orange-red emission. Rather than O1s, we propose a reliable method by considering the peak shift of Zn2p in XPS to inspect the ZnO matrix, which has good interdependence with the characteristics of PL.

## Introduction

Zinc oxide (ZnO) based semiconductor devices has attracted enormous attention among researchers because of its versatile applications in a variety of research fields such as optoelectronics, piezoelectronics, solar cells, and have been reviewed extensively^1–4^. The wide direct band gap (E_g_ = 3.4 eV), and high excitation binding energy (60 meV) makes this material a suitable alternative to GaN (26 meV). Apart from this, low cost, and ease of manufacturing^2–5^ with environmental friendly properties are added advantage of ZnO.

One-dimensional semiconductor nanostructures have high surface-to-volume ratios and exhibit properties that are suitable for novel device applications^6^. Among all the synthesis methods reported so far, the fabrication of ZnO nanorod arrays (ZNR) grown by chemical methods such as hydrothermal method or aqueous solution method is the cheapest and most convenient way^7^. However, ZnO grown by chemical methods always evolve with intrinsic defects such as oxygen (zinc) vacancy, oxygen (zinc) interstitial, and oxygen (zinc) antisite. There are plenty studies about the generation and annihilation of deficiencies via annealing, but the instability properties of self-assembly (SA) ZNRs induce the difficulties on the analyses of surface states and related deficiencies via annealing^8^

The presence of these defects suppresses the ultraviolet (UV) emission efficiency in photoluminescence^8,9^ that degrade the optical properties of ZnO, and inhibit its application in optoelectronic devices. Therefore, it is essential to either grow high quality ZnO nanostructures directly or improve the quality of as prepared ZnO by post synthesis processing. The literature survey reveals that the optical properties of ZnO can be improved by post-annealing treatment at elevated temperature^10,11^ which provides enough activation energy to organize the Zn-O bonds leading to surface reconstruction and rearrangement of oxygen defects.

The effect of annealing temperature, annealing atmosphere^12–20^ on the structure, optical property, and stability of ZnO single crystals/nanostructures/nanorod arrays^17–19^, thin films^14^ have been studied previously. Babu *et al*.^12^, have reported a regular increase in the intensity of green luminescence (GL) with an increase in annealing temperature, which is attributed to the increase of oxygen vacancy in the beginning that saturates at 800 °C, and evaporation of Zn atom at high temperature. Cui *et al*.^14^, have described the effect of annealing temperature and annealing atmosphere (air, argon, nitrogen and oxygen) over ZnO thin films fabricated by sputtering, and to determine the optima annealing condition. Ruqia *et al*. conducted an experiment to demonstrate the correlation between aspect ratio and the crystallinity of ZNRs grown by the solvothermal synthesis^21^. They performed UV-Vis absorption spectrum and PL to analyze the recombination of exitons and the existence of defects in ZNRs, which exhibited the reduce of band width and the increasing signal of visible emission. Non-well-crystalized ZnO annealed in oxygen atmosphere with the increase in the annealing temperature may raise the oxygen vacancy concentration^22,23^, however, it is very difficult to understand whether as annealing in oxygen atmosphere would compensate oxygen vacancies in ZnO or not. Therefore, we strongly believe that the first few layers over the surface of ZNRs fabricated by wet chemical synthesis consists of unstable surface defects, surface dangling bonds, precipitates/segregations/organic residues and surface OH¯ ions that virtually suppress all signals from the intrinsic defects of ZnO. This unstable surface structure leads to a large inconsistency in the data obtained from optical analyses by PL with the surface properties obtained by XPS of as prepared and annealed ZNRs.

For SA-ZNRs, the annealing process at any condition actually create lots of inconsistent data in the results of optical analysis which is not enough explain all aforementioned issues. This may be due to the limited facility available with the annealing systems and limitations with the analytical tools for measurement. This gives us an additional impetus to study the surface properties of ZNRs in more detail, and to build a mechanism for the surface modification of ZNRs during annealing.

In order to really realize the influence of oxygen atoms for the intrinsic defects of SA-ZNRs and further comprehend the evolution of surface states of ZNRs during annealing, ZNRs with stable structure and surface states is necessary. Here, we propose a standard two step annealing process, where the ZNRs are subjected to the thermal treatment in the vacuum (first step) at 800 °C for 20 min to remove (create) unstable surface defects (stable structural configuration), and, then, in oxygen atmosphere at the same condition (second step) to create intrinsic defects. The surface properties are analysed extensively by photoluminescence (PL) as well as X-ray photoelectron spectroscopy (XPS) measurements and a strong correlation is established between these two measurements. Usually, the core level oxygen (O1s) in XPS spectrum is regarded as the standard for the investigation of surface properties of oxygen in ZnO. However, O1s in XPS spectrum can describe the variation of oxygen vacancies, but it cannot distinguish oxygen interstitials, zinc vacancies. Therefore, in addition to the optical analysis by PL, the combined analysis of O1s, and Zn 2p in XPS spectrum can reveal more information about the evolution mechanism of surface structure and defects in ZNRs.

## Results and Discussion

Before examining the influence of double annealing effect on ZNRs in vacuum/oxygen atmosphere, one step annealing experiments at different conditions are done and the surface properties are analyzed by PL and XPS.

### Defect formation in ZnO after one time annealing

**Figure 1:**
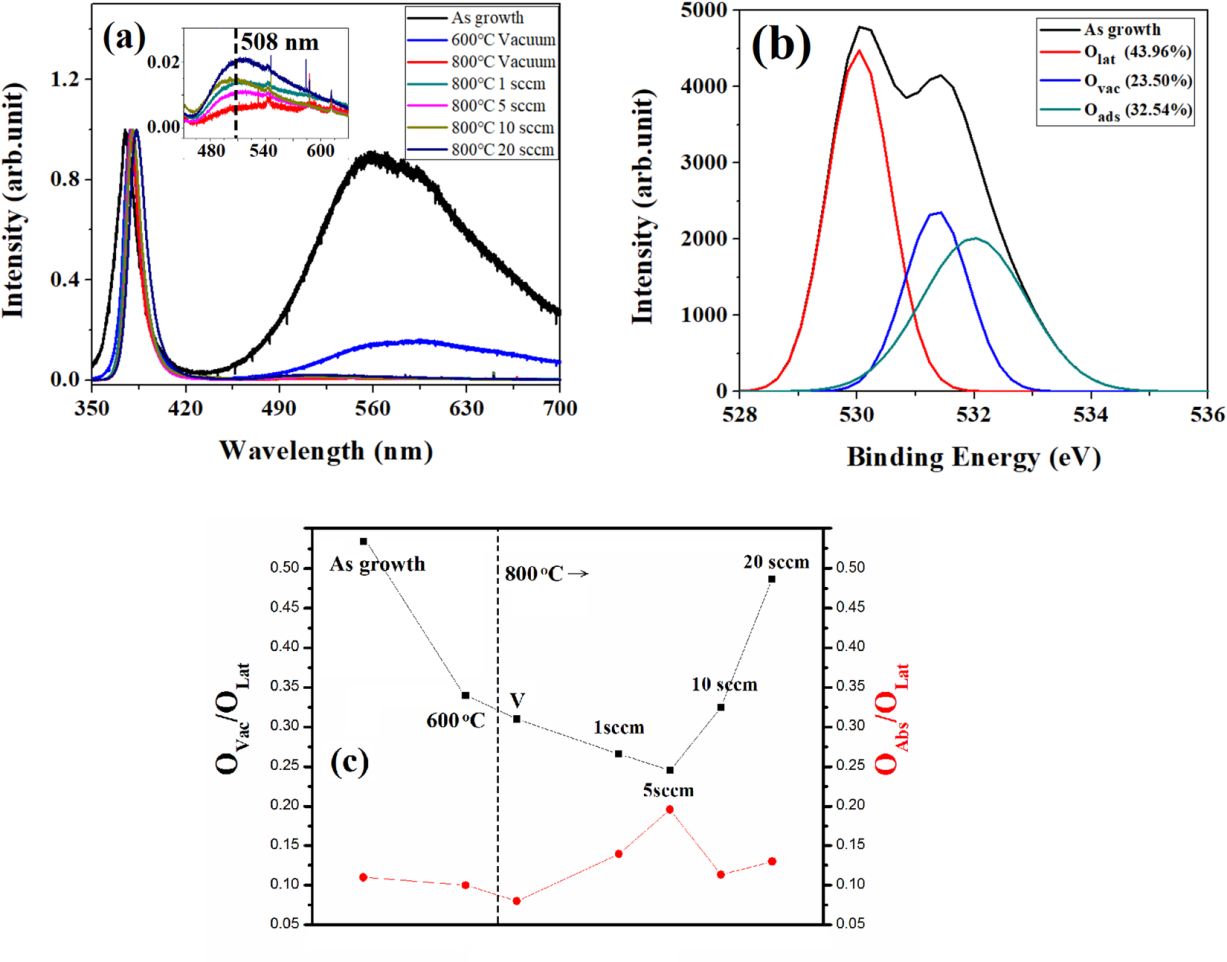
(**a**) PL spectrum for one time annealing with different temperature and oxygen pressure. Inserted figure is the enlarged PL in the visible luminescence region. (**b**) O1s XPS spectrum for as growth ZNRs with VA process. The spectrum is deconvoluted to three specific peaks: the Zn-O bond in ZnO matrix (O_Lat_.) centered at 530 eV, the oxygen vacancies (O_Vac_) centered at 531 eV and the oxygen chemical adsorption (O_Ads_) centered at 532 eV. (**c**) Trend of O_vac_/O_lat_ and O_Ads_/O_lat_ in XPS spectra of O1s for ZNRs annealed with the different temperature and oxygen pressure.

The room temperature PL spectra (Fig. 1(a)) of single annealed ZNRs shows two distinct peaks: (a) A strong UV emission originating from the recombination of free excitons and is considered as the characteristic signature of wurtzite ZnO, (b) the visible luminescence centered at 558 nm is usually attributed to all possible surface defects dominated by OH^−^ ions produced usually in ZnO grown by wet chemical method^24^. This peak assignment is doubtful and we repeat that the position/broadness of the peak may be related to the unstable surface defects generated during synthesis, surface dangling bonds, precipitates/segregations/organic residues and surface OH^−^ ions that virtually suppress the intrinsic defects in ZNRs. The visible luminescence intensity decreases after annealing at 600 °C with the peak still centered at 558 nm. However, the peak is completely quenched at 800 °C, but, a new peak (inset in Fig. 1a) at 508 ± 8 (2.44 ± 0.33 eV) nm is emerged corresponding to a clear, distinct, intrinsic green luminescence (GL) in ZnO. This peak position is much closer to that obtained previously (2.52 eV) in ZnO annealed at Zn rich condition and assigned to oxygen vacancy (V_o_)^25^. The annealing energy at 600 °C is not high enough to remove the loosely bound surface defects in ZNRs and 800 °C annealing in vacuum seems to provide sufficient energy to eliminate all such defects thereby making GL emission clearly visible. The position of GL emission peak at 508 ± 8 nm does not change much for ZnO annealed at 800 °C irrespective of the oxygen flow rate. It is interesting to note that the intensity of the newly emerged GL peak at 508 nm is the lowest for ZnO annealed in vacuum and that increases with the increase in oxygen flow rate with a minor fluctuation in the intensity at 1 sccm oxygen flow rate. Generally, annealing of ZnO in oxygen atmosphere would compensate the originally created oxygen vacancies and decrease the intensity of GL emission and therefore, the increase in the intensity of GL emission peak with the increase in oxygen flow rate is quite intriguing.

O1s in XPS is used to inspect the chemical state of ZNRs where the spectrum is deconvoluted to three specific peaks: the Zn-O bond in ZnO matrix (O_Lat._) centered at 530 eV, the oxygen vacancies (O_Vac_) centered at 531 eV and the oxygen chemical adsorption (O_Ads_) centered at 532 eV^26–28^. For example, the deconvoluted O1s XPS of as growth ZNRs is shown in Fig. 1(b).

Figure 1(c) shows the corresponding area ratios of O_Vac._/O_Lat._ and O_Ads._/O_Lat_ with annealing temperature and oxygen flow rate. The primary O1s spectra for one-step annealing are shown in Supplementary Information Fig. S1. The O_Vac._/O_Lat_ ratio decreases gradually upto 5sccm oxygen flow rate and that increases with increase in oxygen flow rate from 5 sccm to 20 sccm. O_Vac._/O_Lat_ ratio is much higher for ZNRs annealed at 800 °C in vacuum which is in contrast with the PL observation that shows lowest intensity GL emission (oxygen vacancies concentration is minimum). The trend of O_Vac._/O_Lat_ and O_Ads_./O_Lat_ shows exactly opposite behavior with increase in the oxygen flow rate. This may be due to the formation of different defects on the surface and related adsorption of various species (O_2_, OH^−^, CO, CO_2_ etc.) on the surface in ambient and that makes the study very complicated.

In order to overcome the unstable and complex configuration of ZNRs, we have considered two step annealing process where the ZNRs are annealed in vacuum (VA) followed by oxygen (OA) atmosphere (VA-OA process) and vice versa (OA-VA process). We expect that the ZNRs after annealing in VA process would remove all loosely bound surface defects, surface dangling bonds and produce an ideal ZnO material for the investigation of the influence of oxygen flow rate during second round of annealing.

### The defect formation in the VA-OA process

**Figure 2:**
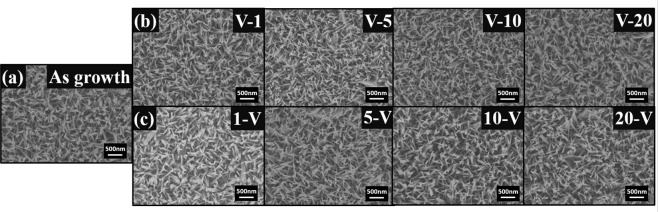
SEM images of (**a**) as growth ZNRs (**b**) ZNRs annealed with VA-OA process 800 °C with oxygen flow of 1 to 20 sccm (**c**) ZNRs annealed with OA-VA process 800 °C with oxygen flow of 1 to 20 sccm.

In order to confirm the suitability condition of the VA process, we have performed PL measurements for SA-ZNRs annealed at 800 °C for 10, 20 and 30 min (see Supplementary Information Fig. S2). It is obvious that ZNRs annealed for 20 min has less visible luminescence intensity. The result demonstrates the minimum deficiencies in ZNRs annealed at 800 °C for 20 min. The condition of the annealing at 800 °C for 20 min is treated as “VA” process in this work. Besides, SEM images for as-growth ZNRs, ZNRs via VA-OA process and ZNRs via OA-VA process are exhibited in Fig. 2. ZNRs annealed with VA-OA or OA-VA process keeps similar morphologies as as-growth ZNRs.

**Figure 3:**
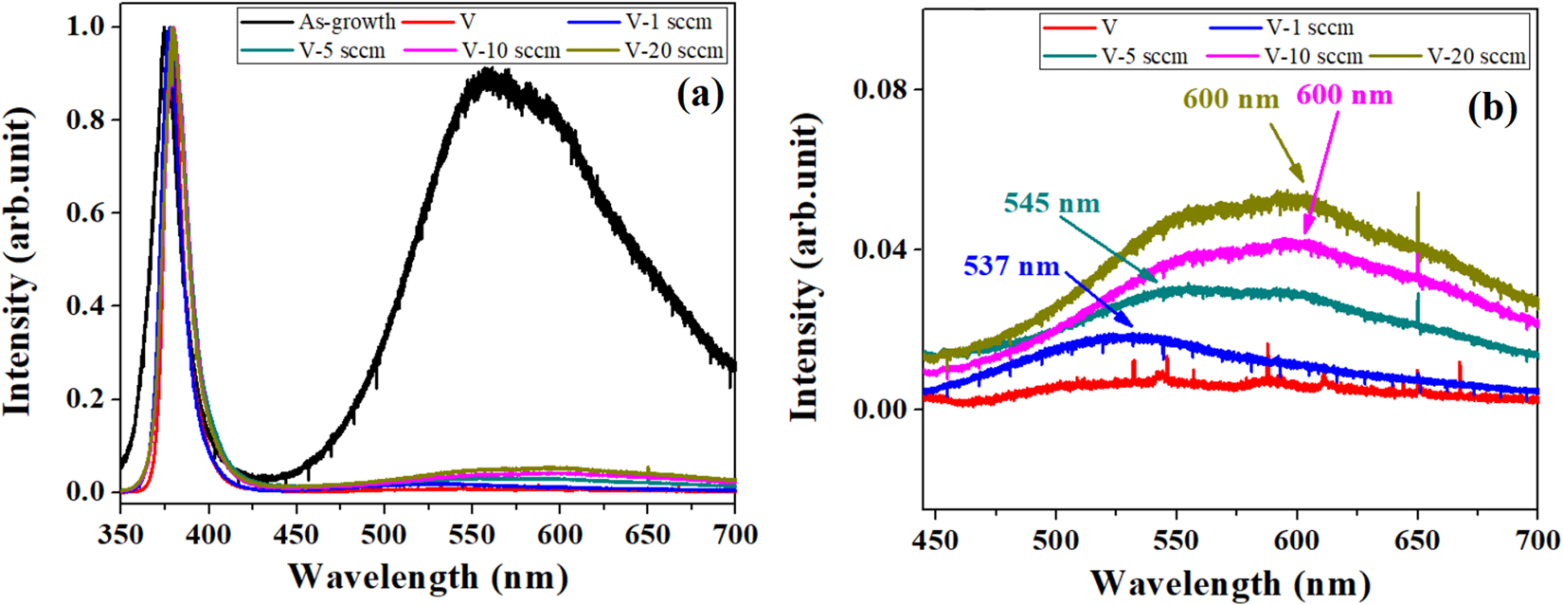
(**a**) PL spectrum of ZNRs annealed with the VA-OA process (**b**) enlarged PL spectra in visible luminescence region.

Figure 3 shows the photoluminescence (PL) spectra of ZNRs after VA-OA process where the ZNRs are annealed in vacuum for 20 minutes and then with oxygen flow rate for another 20 minutes. As before, the as grown ZNRs show two major peaks: one at ~379 nm corresponding to the characteristics UV emission and the visible luminescence is centered at 557 nm (Fig. 3a). Similar to one step annealing process, the visible luminescence peak at 557 nm is completely quenched and the a new peak at 508 nm is observed for ZNRs annealed at 800 °C in vacuum. Subsequent samples subjected to VA-OA process shows a regular increase in the intensity of GL emission centered at 537 ± 10 nm (2.30 ± 0.4 eV). The peak position also agrees well with the peak (2.30 eV) obtained previously^25^ for ZNRs annealed at oxygen rich conditions and assigned to zinc vacancy. The orange-red emission centered at 600 nm (2.06 eV) is caused by the oxygen interstitials (O_i_) (Fig. 3b) produced during the OA process where the oxygen atoms dissociate and diffuse into the surface of ZNRs. These results reflect the strong dependence between the extrinsic oxygen atoms and formation of excess O_i_. Although, the formation of oxygen interstitial related orange-red emission is reasonable in the present context, the increase in the green emission intensity is explained by the combined analysis of Zn 2p and O1s in XPS spectra.

**Figure 4:**
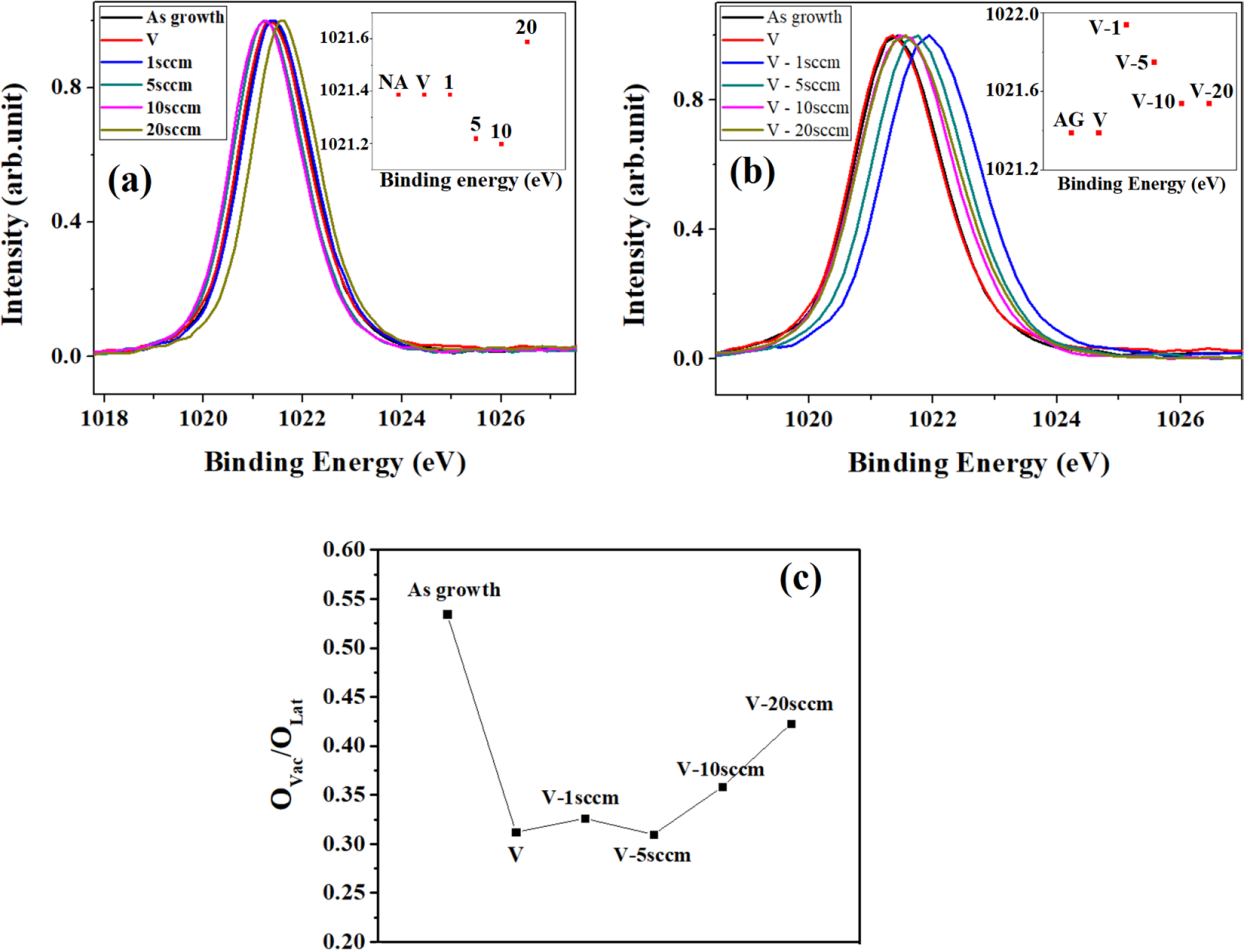
(**a**) XPS spectra for Zn2p of ZNRs annealed with the one-step annealing process and inserted figure is the trend of the binding energy of Zn 2p. (**b**) Zn 2p in XPS spectra for ZNRs annealed with the VA-OA process, and inserted figure is the trend of the binding energy of Zn 2p for as growth ZNRs (AG), the oxygen flow of 1 sccm (V-1), 5 sccm (V-5), 10 sccm (V-10) and 20 sccm (V-20) in the VA-OA- process. (**c**) Trend of O_vac_/O_lat_ in XPS spectra of O 1s for ZNR annealed with the VA-OA process.

Before examine Zn2p XPS of VA-OA process, we performed Zn2p XPS of SA-ZNRs treated with one-step annealing to confirm the unstability of SA-ZNRs, as shown in Fig. 4(a). The trend of Zn2p XPS with oxygen pressure is inserted in Fig. 4(a). It is obvious that the trend of Zn2p with oxygen pressure is not regular in the one-step annealing due to the unstable surface structure of SA-ZNRs. Figure 4(b) shows Zn 2p in XPS spectra where the Zn 2p peak position shifts from 1021.43 eV for as prepared ZNRs to 1021.34 eV for ZNRs annealed at 800 °C in vacuum. These peak positions indicate that the surface of as prepared and vacuum annealed ZNRs is Zn dominated (1022 eV for stoichiometric ZnO). This implies that the surface of as prepared ZNRs is zinc rich with high concentration of oxygen vacancy and that increases after annealing in vacuum. The Zn 2p peak position shifts to high binding energy (1021.93 eV) for ZNRs annealed at 1sccm oxygen flow rate and further increase in the oxygen flow rate leads to shift toward lower binding energy (1021.75 eV at 5 sccm, 1021.55 eV at 10 (20) sccm oxygen flow rate). This indicates a gradual modification in the surface configuration of ZnO matrix from well structured ZnO to Zn rich condition. This further suggests the formation and increase in the oxygen vacancies with the increase in the oxygen flow rate and that agrees well with the PL observation. This is also evident in Fig. 4(c) (area ratio O_Vac_/O_Lat_ obtained from O1s in XPS) that shows high oxygen vacancy concentration for as prepared ZnO, low oxygen vacancy for ZNRs annealed at 1sccm oxygen flow rate and a regular increase in the area ratio of O_Vac._/O_Lat_ with the increase in the oxygen flow rate (5–20 sccm). The primary O1s spectra for VA-OA annealing are shown in Supplementary Information Fig. S3. However, the vacuum annealed ZnO does not show high oxygen vacancy in O1s spectrum, which may be due to the fluctuations created by the surface adsorption. In XPS spectra, the oxygen deficiencies such as vacancies, interstitials, and antisites are not distinguishable and therefore, it is not possible to analyze all the defects to evaluate the influence of vacuum and oxygen flow rate during the annealing process. With measuring the Zn2p XPS, the optical response of ZnO lattices which correlated to the signal centered at 530 nm in O1s spectra could be further examined. The discrepancy on the analysis of O1s XPS, which only measured the ratio of O_lat_/O_vac_/O_ads_, would be solved by exactly defining the optical response of O_lat_ with Zn2p XPS. As the result, a combined analysis of Zn 2p with O1s in XPS is a proper way to analyze the surface and bulk structure of ZNRs.

### The defect formation in the OA-VA process

**Figure 5:**
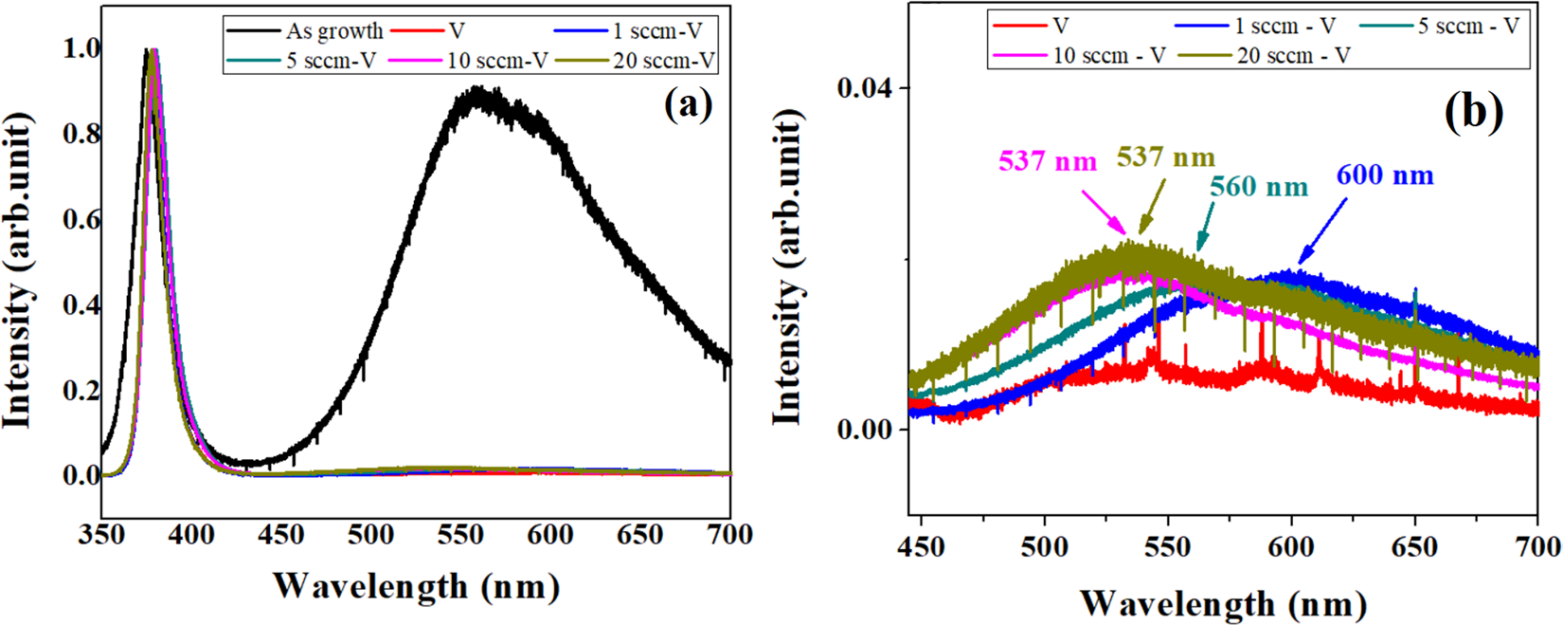
(**a**) PL spectrum of ZNRs annealed with the OA-VA process (**b**) enlarged PL spectra in visible luminescence region.

We also performed the OA-VA annealing process on SA-ZNRs to examine the effect of reverse annealing process over the stoichiometric changes in the surface/bulk configuration of the as prepared ZNRs. Figure 5(a) shows the PL spectrum of ZNRs annealed with OA-VA process at 800 °C. A careful observation of the visible part of the PL spectra (Fig. 5(b)) reveals gradual decrease in the intensity of the orange-red emission (600 nm) and regular increase in the green-red luminescence (537 nm). After the first OA process, solid O_i_, O_Zn_ defects are generated so that these residual O_Zn_ and O_i_ defects (the peak of visible luminescence at 537 and 600 nm) could be kept in the following VA process. The formation energy of O_i_ defects depends on the location in the ZnO matrix and its value varies from 1.42 eV to 2.07 eV^29,30^. After sequent VA process, some of the loosely bound O_i_ defects over the surface would be eliminated and the others would form octahedral O_i_ (O_i, oct_) with high binding energy^30–32^ which contribute toward the orange-red emission at 600 nm. The origin of green luminescence at 537 nm (2.30 eV) along with the increase in the intensity for ZnO prepared in oxygen rich condition is attributed to the presence of V_zn_ or V_zn_ related complexes^25^.

**Figure 6:**
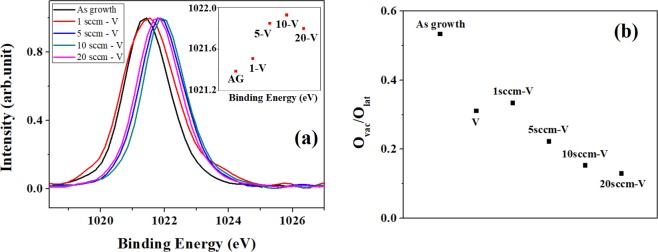
(**a**) Zn 2p in XPS spectra for ZNRs annealed with the OA-VA process, and inserted figure is is the trend of the binding energy of Zn 2p for as growth ZNRs (AG), the oxygen flow of 1 sccm (1-V), 5 sccm (5-V), 10 sccm (10-V) and 20 sccm (20-V) in the OA-VA- process. (**b**) Trend of O_vac_/O_lat_ in XPS spectra of O 1 s for ZNR annealed with the OA-VA process.

The analysis of Zn 2p in XPS on ZNRs treated with the OA-VA process confirm the results of PL spectra and distinguish the difference of the restructured ZNRs between the OA-VA and VA-OA process. Figure 6(a) shows the Zn 2p in XPS spectrum of ZNRs after the OA-VA process where the peak position gradually shifts to 1022 eV, which represents that the ZnO surface is gradually modified from Zn rich condition to stoichiometric ZnO. However, the pattern seems to shift again toward Zn rich condition without having any change in peak position for ZnO annealed in 20 sccm oxygen flow rate-vacuum and the shift is not that significant to make any specific conclusion. The Zn 2p in XPS spectrum for ZnO annealed at 1–10 sccm oxygen flow rate followed by annealing in vacuum shows a gradual modification of surface from rich Zn to stoichiometric ZnO. The shift of Zn 2p toward low binding energy for ZnO annealed at 20 sccm oxygen flow rate and vacuum gives an indication about the formation of oxygen vacancies which agrees well with the evolution of green luminescence of the same ZnO sample. On the other hand, the trend of O 1 s in XPS is shown in Fig. 6(b) which exhibits the evolution of V_o_ during the OA-VA process. The primary O1s spectra for OA-VA annealing are shown in Supplementary Information Fig. S4. The area ratio of V_o_ (centered at 531.0 eV) decreases when the oxygen flow rate in the OA process increases which demonstrates a different result with PL spectrum. The result of O1s in XPS is no clear to clarify the category of deficiencies and even adsorbed O atoms in XPS. The surface states of ZNRs become complicated in OA process because the diffusing and recrystallizing process were disturbing by the extrinsic oxygen atom so that the unexpected, solid defects were induced and then kept in the sequent VA process. Therefore, O1s in XPS for OA-VA process can not match the analyses of PL and XPS for Zn2p.

### Mechanism of defect formation/repair in SA-ZNRs in double annealing

#### VA-OA process

**Figure 7:**
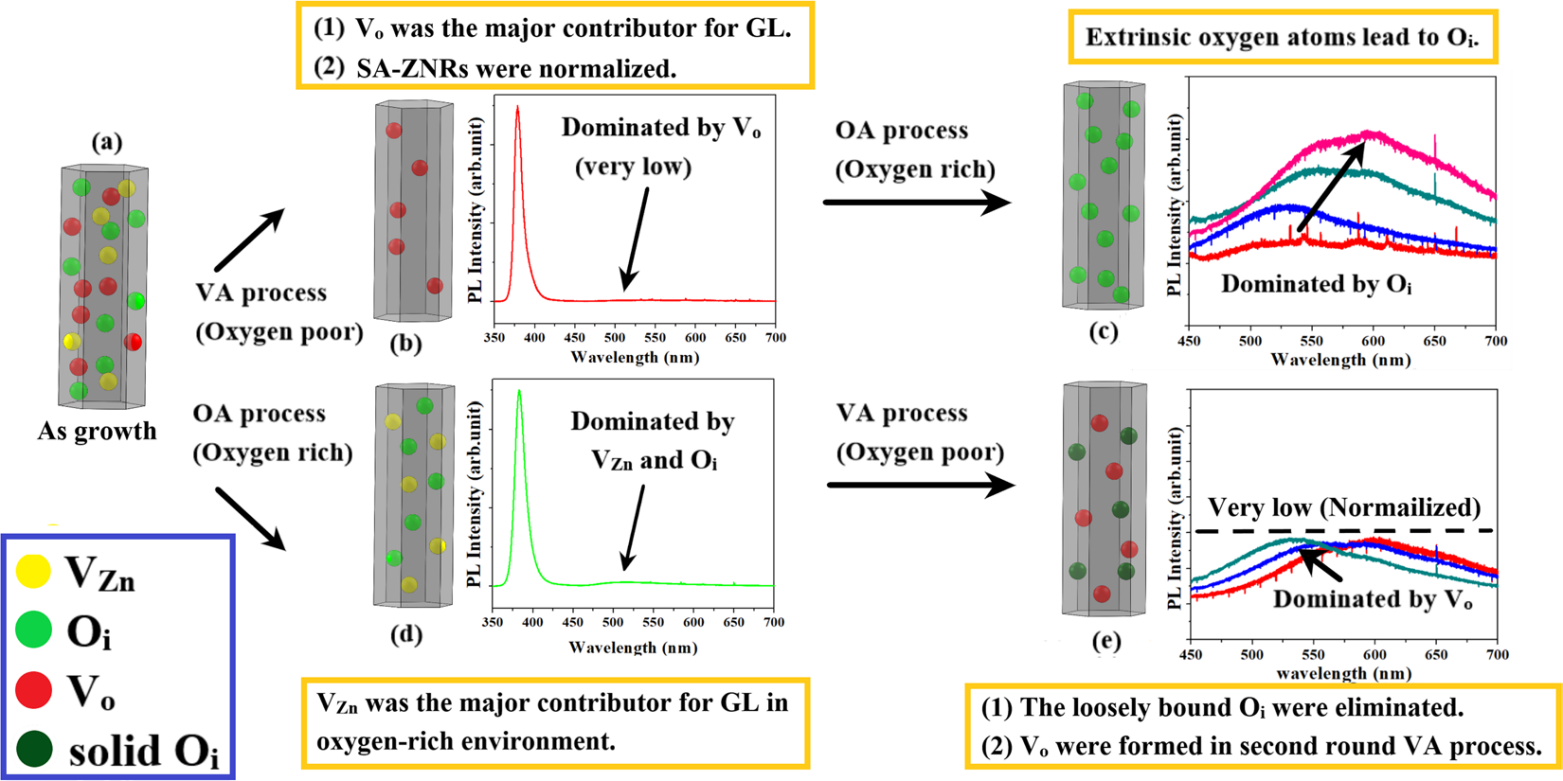
The mechanism diagram of (**a**) AG ZNRs and ZNRs after (**b**) VA process (**c**) VA-OA process (**d**) OA process (**e**) OA-VA process.

We have following observations from our strategic VA-OA annealing process: (a) weak green luminescence with moderate increase in the intensity, (b) strong orange-red emission with regular increase in the intensity, (c) surface of ZnO annealed at high oxygen flow rate is dominated with Zn as observed by Zn2p in XPS, and (d) increase of O_vac_/O_lat_ ratio with increase of oxygen flow rate. The mechanism of deficiencies in the VA-OA process is diagrammed in Fig. 7(a–c).

There are several theoretical investigations about the formation energy of the intrinsic defects in ZnO^33^. The defect formation energy as described by Oba *et al*.^34^, is suitable for our experimental conditions as it describes the formation energy of oxygen vacancy in oxygen poor and oxygen rich conditions. They have found low (high) formation energy of oxygen vacancy in oxygen poor (rich) condition. We also follow one of the experimental data that assigns the GL emission to the zinc vacancy for ZnO annealed in oxygen rich condition^25^. The GL is usually attributed to the presence of V_o_ and V_zn_ as shown in Fig. 7(b,d). Here, the formation of V_o_ in oxygen poor condition and V_zn_ in oxygen rich condition as the major contributor for the GL emission. The increase of oxygen vacancy with the increase in oxygen flow rate from 5 sccm-20 sccm is very intriguing, as high oxygen flow rate would compensate oxygen evaporation from ZnO surface during annealing at high temperature and that would lead to decrease in oxygen vacancy concentration due to incorporation of oxygen. The deficiency formation during VA-OA process is summarily shown in Fig. 7(a–c). Oxygen vacancies are produced during the VA process and majority of these oxygen vacancies are supposed to be repaired and some of them still remain as it is during OA process. Apart from this, zinc vacancies can also be produced in ZnO annealed at oxygen rich condition^25^. So, the increase in the green emission intensity at 537 ± 10 nm may be attributed to the formation of zinc vacancies. This combination of V_o_ and V_Zn_ lead to the increase in the intensity of the green emission. OA process only produces oxygen interstitials and that may lead to relatively strong orange-red emission along with the increase in the intensity. O_Zn_ has much higher formation energy than Oi and its role may not be very significant. The shift of Zn 2p peak in XPS provides the information about the quality evolution of ZNRs and is consistent with PL. Although, we are able to propose the mechanism for the first two effects, the formation of excess Zn over the ZnO surface after VA-OA annealing still needs further investigation.

#### The OA-VA process

The crucial observations obtained from OA-VA annealing process are summarized as follows: (a) strong green emission along with the increase in the intensity, (b) increase in the intensity of the orange-red emission (although very weak). The mechanism of deficiencies in the VA-OA process is diagrammed in Fig. 7(a,d,e). Oxygen vacancy has high formation energy in oxygen rich condition and during OA annealing process, oxygen vacancy either does not form or form with very low concentration, but oxygen interstitials can form in high concentrations, as shown in Fig. 7(d). However, second round of annealing in vacuum would lead to the elimination of the majority of the loosely bound O_i_ and formation of oxygen vacancies and the combined effect of OA-VA annealing process would lead to strong green emission along with weak orange-red emission. During OA-VA process (as shown in Fig. 7(e)), the same theoretical and experimental findings can describe the origin of green luminescence.

Furthermore, zinc vacancies produced in ZnO during OA annealing would not be affected in VA process. This will add up with the oxygen vacancies produced during the VA process and will lead to increase in the green emission intensity. The variation in the intensity of both the emissions as well as the peak shift from orange-red to green-red luminescence is attributed to the competition between the formation/elimination of O_i_, V_o_ and V_zn_ during the OA-VA process. It is still very difficult to explain the origin of orange-red emission for ZnO annealed in 1sccm oxygen flow rate followed by vacuum. This may be due to the formation of the complex defect states and is beyond our scope of investigation.

Besides, The Zn 2p spectrum for ZnO annealed shows a gradual modification of surface of ZnO from rich Zn to stoichiometric ZnO with increase in oxygen flow rate, and shift toward low binding energy for ZNRs annealed at higher oxygen flow rate in the OA-VA process. The simple structure indication from Zn 2p in XPS agree with the evolution of green luminescence in PL for ZNRs.

The defect formation of SA-ZNRs via annealing with different oxygen rate is complicated due to the unstable and complex configuration of SA-ZNRs. The performance of VA-OA provides stabilized ZNRs configurations and appreciable formation energy of defects to further realize the mechanism of defect formation.

For ZnO gas sensors, the intrinsic defects are the key points that dominate the type of adsorption/desorption and the amount of defects which participate in the chemical reactions would directly affect the sensitivity of gas sensors^35–37^. In order to promote the properties of ZnO-based gas sensors, the chemical configurations and the distribution of defects should be clearly realized. In this work, the XPS spectra for O1s and Zn2p were analyzed together to solve the uncertainty of O_lat_ in O1s spectra which exhibited the defect elimination through VA process and the formation of the oxygen species with the OA process in the first 10 nm from the surface. Combining with the PL spectrum, the intrinsic defects in the whole ZNR could be inspected, which can separate the signal of surface and bulk. With the technologies of VA/OA annealing process and the multi-optical analyses, the structural evolution of ZNRs could not only be well controlled but also be accurately monitored.

## Conclusion

In this work, we proposed normalized ZNRs by performing SA-ZNRs in VA process to provide reliable ZNR configuration for further study on the influence of extrinsic oxygen atoms. These series of VA-OA annealing process were used to realize the influence of extrinsic oxygen atoms via the annealing process and the results demonstrated O_i_ defects have a positive correlation with the oxygen flow rate. Furthermore, a reverse annealing (OA-VA) process would assist us to understand the influence of extrinsic oxygen atoms to SA-ZNRs via annealing and further residual or additional defects generated by annealing process with O flow, which sequent VA process will eliminate the defects with weak binding energy.

To realize the element configuration, XPS measurement for Zn 2p is demonstrated rather than XPS measurement for O1s. The single peaks of Zn 2p in XPS spectra gives a clear physical mechanism of the structural changing which represent the environment of Zn atoms on the surface. By inspecting the XPS spectra for Zn2p, the influence of oxygen atoms is estimated with a reliable physical mechanism. In our work, excessive physical species are evolved with oxygen atoms such that the XPS spectra for O1s are difficult to reveal what the influence of each operation and the role of oxygen atoms.

## Methods

### Sample preparation

The ZnO seed layers were deposited on a glass substrate by radio-frequency magnetron sputtering using a ZnO target with 99.99% purity^38^.ZNR arrays were grown on the ZnO-seeded layer by hydrothermal method. The mixed aqueous solution consisted of 25 mM zinc nitrate hexahydrate [Zn(NO_3_)_2_.6H_2_O, 99.0%], and 25 mM hexamethylenetetramine [C_6_H_12_N_4_, 99.0%]. Subsequently, the ZnO-seeded quartz glass substrate was dipped in the mixed aqueous solution at 90 °C for 8 h to grow the ZNRs. After growth, the samples were annealed at 800 °C for 20 min by using a rapid thermal annealing (RTA) furnace (ULVAC MILA-3000) with different oxygen flow rates (in vacuum and 1–20 sccm). The heating rate of RTA was 40 °C/s. The post-annealing process was performed to examine the influence of oxygen flow rate on the crystallization and optical property of ZNRs.

### Characterization

The surface morphology of the samples was characterized by a field-emission scanning electron microscope (FESEM) operated at 5 kV. PL spectra of the samples were measured by using a He-Cd laser (λ = 325 nm) as the excitation source. The chemical composition and valance state of the ZNR surface were examined by XPS (Kratos Axis Ultra DLD).

## Supplementary information

Supplementary information
