# Supplementary material for: Strong correlation between optical properties and mechanism in deficiency of normalized self-assembly ZnO nanorods
Source: Sci Rep. 2019 Jan 29;9:905. doi: 10.1038/s41598-018-37601-8 (PMC6351557; doi:10.1038/s41598-018-37601-8)
Supplement: Supplementary file 1 — Supplementary information [file 41598_2018_37601_MOESM1_ESM.pdf]

Supplementary Information

Article in *Scientific Reports*

Strong correlation between optical properties and mechanism in deficiency of normalized self-assembly ZnO nanorods

Feng-Ming Chang, Sanjaya Brahma*, Jing-Heng Huang, Zong-Zhe Wu, Kuang-Yao Lo*

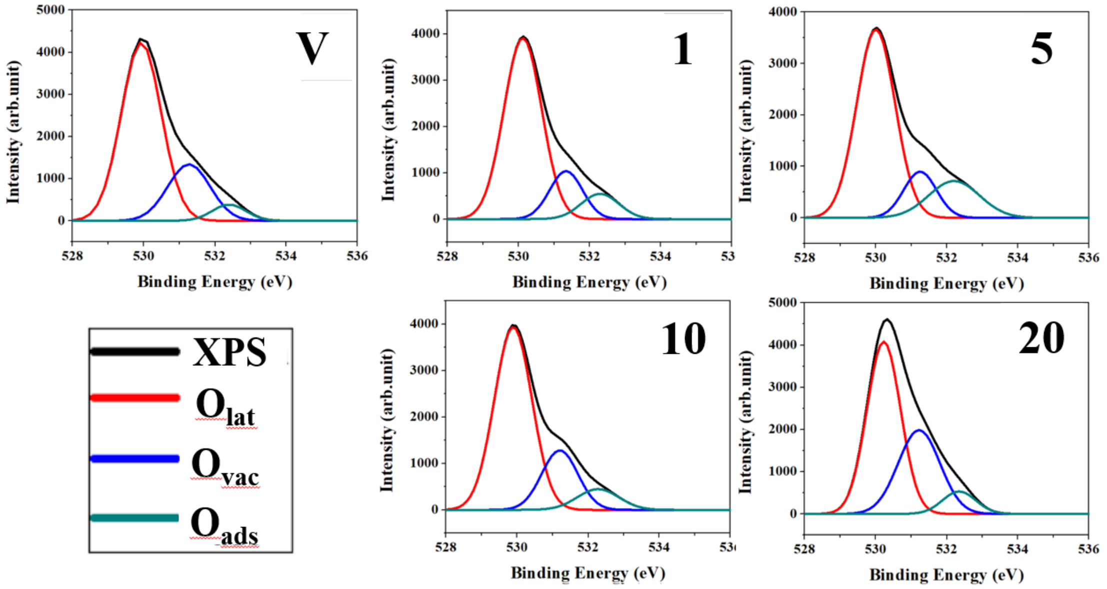

Figure S1. XPS spectra for O1s of single annealed ZNRs.

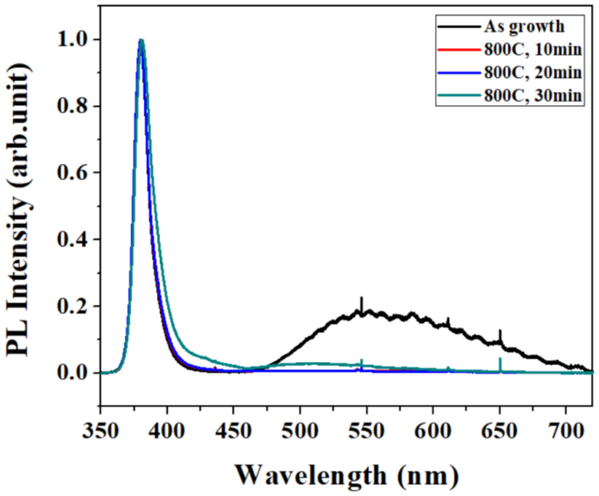

Figure S2. PL spectrum of ZNRs annealed at 800°C for 10/20/30 min.

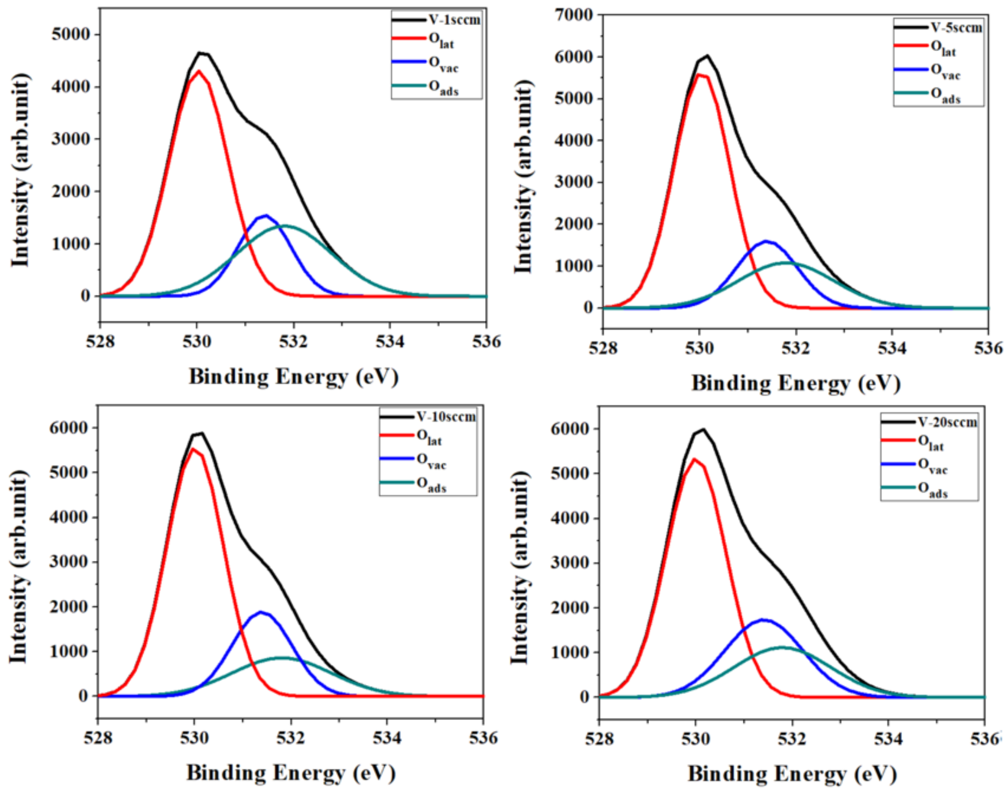

Figure S3. XPS spectra for O1s of ZNRs annealed with VA-OA processes.

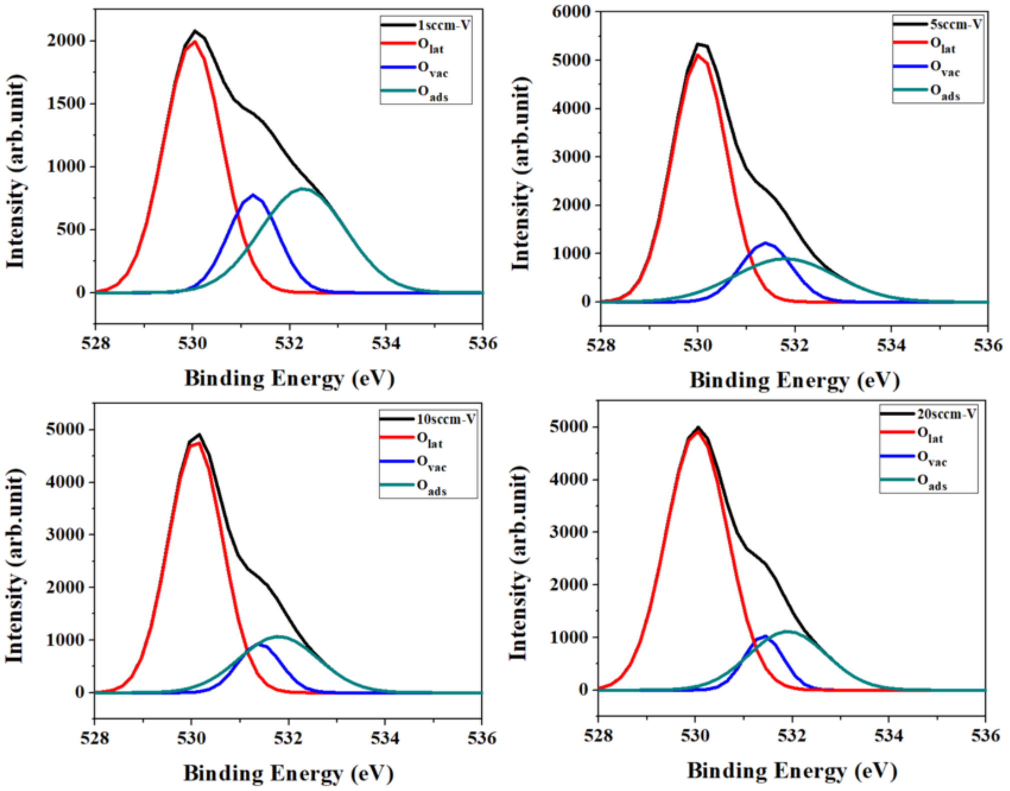

Figure S4. XPS spectra for O1s of ZNRs annealed with OA-VA processes.
